# The Disjointed Historical Trajectory of Anorexia Nervosa Before 1970

**DOI:** 10.1007/s11920-015-0641-6

**Published:** 2016-01-14

**Authors:** John P. M. Court, Allan S. Kaplan

**Affiliations:** Centre for Addiction and Mental Health, Department of Psychiatry, Institute of Medical Science, Faculty of Medicine, University of Toronto, 1001 Queen Street West, Toronto, ON M6J 1H4 Canada; Graduate and Academic Affairs, Faculty of Medicine, Campbell Family Mental Health Research Institute, Centre for Addiction and Mental Health, University of Toronto, Toronto, ON M5T 1R8 Canada; Eating Disorder Community Treatment Program, Toronto General Hospital, Toronto, ON M5G 2C4 Canada

**Keywords:** History of psychiatry, Eating disorders, Anorexia nervosa, Psychiatry, Neurology, Psychotherapy

## Abstract

Responses in pre-modern eras to anorexia nervosa (as now understood) varied widely, from religious piety and sanctity through fear and superstition. While noting briefly the limited conceptualizations from pre-modern history this article is primarily focused from the late 19th century, commencing with helpful but tentative formulations of anorexia nervosa for early-modern medicine that were laid out, consistently between themselves, by Lesègue, Gull and Osler. Yet that promising biomedical advent was superseded for more than a half-century by deep, internal divisions and bitter rifts that festered between three medical disciplines: neurology; Freudian psychotherapy; and Kraepelinian biological psychiatry. Mid–20th century developments preceded the 1960–1980s’ improved understanding of suffering and movement toward effective remediation introduced by Dr. Hilde Bruch.

## Introduction

Helping sufferers, their families and their health care providers to have an understanding of their ailment or disorder is an essential first step toward overcoming it. Eating disorders (EDs^1^), and specifically anorexia nervosa (AN), have long been characterized by unusually staggering complexity, even in comparison with other intractable functional (inorganic) mental disorders. This is the case from every aspect—origins and cause, nosology, their immediate physical and emotional effects, their long-range impact on sufferers’ and their families’ health and functioning, the treatment options and prognosis for recovery. An understanding of AN’s historical evolution offers an accessible, complementary perspective for sufferers and their families—in conjunction with scientific explanations, current treatment approaches and their prognoses.

History affords a critical perspective for assessing AN on account of it being consistently encumbered by both conceptual and clinical ambiguities that interfere with effective diagnosis and efficacious treatment. The specific factors of these ambiguities have long included: a (varying) persistence of stigma; a lack of clinical legitimacy, exacerbated by non-physiological explanations for its origin/ aetiology; a continuing uncertainty regarding effective treatments; and an unpredictability of the nature of the illness-course and of its outcome. Only in recent decades have those issues begun to diminish, yet reliable explanations for this improvement remain elusive, and vestiges of them continue to affect patients and caregivers, particularly when paired with systemic barriers to assessment and treatment such as long waiting times for in-hospital care.

Those in past Western societies afflicted with AN (as now understood) were assessed, if at all, in terms of one or more prevailing epistemologies of their time—physical healing, spirituality or superstition. They were then offered remedies or dispositions that would now be considered for the most part as notably less effective than those for their modern counterparts who, in the most recent era commencing with the approach of Dr. Hilde Bruch (1904–1984), have benefited from biological psychiatry in conjunction with variants of post-Freudian psychotherapy [1, 2].

Bruch’s compelling fundamental principles, grounded in her charismatic yet humble humanity, led to a solidifying of organized medicine’s conceptualization of AN within the field of psychiatry. This consensus was a contrast to notable departures by certain previous modern psychiatric approaches or specific treatments, when illness classifications were removed from psychiatry’s ambit, for lack of a conceptual fit or success in remediation. Notable examples include organic degenerative brain disorders, such as epilepsy, which were transferred to neurology. Eating disorders and specifically AN were among the few within organized medicine that underwent a transition into the ambit of psychiatry. Over the most recent 50 years the transition to psychiatry has led to generally more positive outcomes for those who should benefit—the sufferers.

## Current State of the Art

Doing justice to, or even synopsizing, the present-day medical science and art of treating AN is beyond the scope of this historical review. New medications, therapies, clear evidence of brain dysfunction through neuroimaging genetics and other biomedical advancements, conjointly treated with the new psychotherapies, are offering tremendous new hope.

As one example, although still as-yet unproved and quite experimental—front-page news in Canada’s largest-circulation newspaper during March, 2015 reported, in conjunction with a TV documentary airing that day, on: “How deep brain stimulation helped one woman fight anorexia” -

“I just wanted to die,” recalls this individual, aged 39. “I was just getting to the point (that) I can’t handle this anymore. There’s nothing for me here to live for anymore.” She even began planning her own funeral…. “It just looked like she was going downhill and was not going to ever recover and was going to die in the fairly near future” recalls [Dr. Blake] Woodside [the patient’s UHN psychiatrist]. “She was happy to try the treatment because there was no other alternative for her.” ...

“More than three years have passed since she underwent the DBS [Deep Brain Stimulation] procedure, and the patient’s life has changed dramatically for the better. She is no longer in and out of hospital. Her weight still fluctuates but it doesn’t drop to the dangerous levels it has in the past.

“I’m not saying that it’s fixed everything but it definitely fixed a lot,” she says, now able to help out a lot more on the family farm where she lives. “And it’s definitely given me a bright outlook on life again.” “She feels that she’s got a reason to be alive and is less troubled by things like normal body weight than she had ever been in her life,” says Woodside.

“Seventeen other patients have now taken part in the DBS study and the results are mixed, with improvement in some cases in associated psychopathology but not in core symptoms of AN. But [neurosurgeon Dr. Andres Lozano] suggests caution. “DBS is not a cure,” he says. “We think that this is a symptomatic treatment. It helps the symptoms of the illness, but the illness is probably there, waiting to come back at any second as soon as the stimulation stops.” Still, as far as [the patient] is concerned, without the DBS she’d either be back in hospital or dead.

“I wouldn’t be standing here today. I got my life back” [3].

We emphasize, however, that DBS is just one example of an experimental intervention. Particularly being invasively surgical, and inaccessible to most sufferers currently, it still lacks rigorous evidence and evaluation through randomized placebo (sham) controlled clinical trials in order to support its efficacy.

The search for understanding, preventing and overcoming AN as a phenomenon has long ranged beyond science and medicine, to domains such as spirituality and abnormal behaviour. As with the broader paradigm, AN’s origins, prevention and cures remain enigmatic — despite a substantial literature of case studies, clinical research and narrative history, they remain just out of reach, persistently mysterious. Focusing interest on the history of mental disorder adds perspective for understanding the wider context of the medical-psychiatric mission [4].

## Ancient and Pre-modern Western Civilizations

Perplexed by irrational behaviour, and symptoms unlike those of known disease entities, ancient healers groped for some familiar context that offered an explanation, and possibly a direction to intervene. For many, their closest context appeared to lie either in the realm of “madness” or in that of supernatural aberrations: extreme piety, or diabolical possession as its evil opposite.

The earliest recorded therapeutic approach to mental disorders in Western medicine appeared with the Pythagorean school, notably Hippocrates who described clinical states that resemble psychosis, and problems with impulse control and depression. He understood mental disease to originate not through external, supernatural forces, but from within, owing to an internal imbalance between the four main bodily fluids —blood, yellow bile, black bile and phlegm. It was an accumulation of black bile that led to melancholia and other mental illness.

While those categories of mental illness were confidently accounted for, AN was seldom observed and not well-documented. One source ambitiously places AN-equivalent descriptions “in ancient Egyptian hieroglyphics, Persian manuscripts, scrolls originating in early Chinese dynasties [and] African tribal lore. … [Among the latter,] Some were healed by shamans who induced trance states similar to what we now know as hypnotherapy” [5•].

The saving grace of religious piety as an interpretation for AN-like behaviour in the Western medieval era was that those sufferers were generally spared from the vastly more-prevalent superstitious belief in witchcraft, which carried dire, fatal consequences, primarily for women. Albert Deutsch estimated that more than 100,000 were executed as witches from the mid-fifteenth century until the Age of Enlightenment began to take hold in the eighteenth century [6]. The suffering caused by beliefs in both witchcraft and “holy anorexia” [7] carried tragic impacts.

As with the tragic accounts of religiously inspired anorexic self-sacrifice, a small number of pre-modern bio-medical cases have been traced and documented. They began with the physician who, late in the seventeenth century, described the first-known case of the illness later described as AN. Dr. Richard Morton’s conception was drawn from two of his patients, one a young woman who eventually died despite various treatment efforts, and another a young man whose outcome is ambiguous [8•]. Although Morton was responding to no known epidemic or cluster, his illness formulation saw past the incidences of religious piety and the pandemic superstitions concerning witchcraft, unlike so many of his contemporaries accused by Deutsch of horrific complicity.

Although a few eighteenth and nineteenth century case reports resembled those of Morton, the pre-modern era’s major advance came about in 1873–1874. Two independent yet corroborating formulations emerged in that 1-year period when anorexia nervosa (AN) was identified as we now understand it, and conceptualized as a disorder, by Drs. Charles Lasègue of Paris and Sir William Gull of London [9]. Once again, there seems to have been no spike in outbreaks at that time, and none followed immediately after. Consequently, little notice seemed to follow their writings on AN. Gull had been elevated to the Baronetcy by Queen Victoria for unrelated services. His biographer simply notes that: “His paper on anorexia broke new ground”; however, “his imperious, often sarcastic manner, and dogmatism alienated many colleagues. He was never made president of the Royal College” [10]. The scarcity of clinical and academic interest in AN was no doubt due in part to its perception as a disorder of females (e.g. usually associated with amenorrhea), whose lives were routinely undervalued in the pre-modern eras.

## Modern Medicine’s Century of Discord on Anorexia Nervosa

I (JC) maintain that the foremost reason that this disorder remained marginalized, despite periodic case accounts, lies significantly with organized medicine’s deep divisions and bitter rifts in the century after Lesègue and Gull, in three directions: neurology versus Freudian psychotherapy versus asylum-based “alienism” that shortly evolved as (Kraepelinian) biological psychiatry.

Much like the disjointed continuity from Morton two centuries earlier —sporadic and isolated —the formulations of Lasègue and Gull piqued only limited interest in influential circles, commencing with that of Jean Martin Charcot at the Sâlpetrière. This eminent neurologist recognized through exploring hysteria that AN’s diverse symptoms and mysterious depredations on young patients lay in the realms of both psyche and soma, and could thus be treated through suggestion, particularly via hypnotism. Ernest Jones described the 1885 impact on Charcot’s young student, neurologist Sigmund Freud: “Now, thanks to Charcot, [hysteria] became, almost overnight, a perfectly respectable disease of the nervous system … one that could be the subject of serious study. To use a modern colloquialism, Charcot had ‘put hysteria on the map’” [11].

By the mid-1890s, Freud had published his controversial view that “all neuroses represent a general disturbance of the sexual functions; that the *actual neuroses* (neurasthenia and anxiety neuroses) result from a direct chemical or toxic disturbance, while the *psychoneuroses* (hysteria and compulsion neuroses) represent the psychic expression of these disturbances”[12]. From 1891 to 1914, Freud’s vast canon ultimately included nine titles (Table 1) referencing anorexia [13] as well as case reports of patients with that symptom such as the totemic “Anno O”. Brenda Maddox later observed: “Working alone, Freud saw himself developing a ‘psychology for neurologists’, and in 1896 he began calling it ‘psychoanalysis’” [14•].

**Table 1:** Sigmund Freud’s References to *Anorexie* appear in Nine Publications, 1891 to 1914

Anorexie (9)				
bei einer hysterischen Anorexie.	5	22 a	Psychotherap	1904
die Sexualablehung durch Anorexie ausdrūckt; man wird	12	141 a	Inf Neurose	1914
dauemdes Erbrechen und Anorexie bis zur	1	82 b	Hysterie	1895
aber ich kann mich auf die Anorexie der Frau v. N… als	1	145 b	Hysterie	1895
an soll die Anorexie erst ihre volle Höhe	19	317 b	Fall Nina 2	1893
Grund zu einer hysterischen Anorexie mit Erbrechen gelegt.	19	316 b	Fall Nina 2	1893
sei sehr wechselnd, meist Anorexie. Nie Globen, häufig	19	314 b	Fall Nina 1	1891
Symptome der Hysterie ist Anorexie und Erbrechen. Ich	19	188 a	Mech Hyster	1893
solchen Abulie bietet die Anorexie unserer Kranken. Sie	1	144 b	Hysterie	1895

Legend for each line: The quotes before and after the index word, *Anorexie*, offer context; the numbers following the index word are (left to right) the vol. no. and page no. in the *Gesammelte Werke* of Sigmund Freud; followed by an abbreviated title for the essay, and its date. SOURCE: per endnote 13

Freud’s mind-not-brain approach to mental illness, however, discouraged North American neurologists and psychiatrists from adopting psychoanalysis as a treatment —particularly for the psychoses and other severe, intractable disorders such as AN. Moreover psychoanalysis became dogged by controversy that Freud aroused and sustained throughout the ensuing decades of that prurient age in Western society, largely through insisting that early pre-sexual and other developmental issues in children lay at the root of many neuroses and psychological disorders. In the early twentieth century, taking the Toronto medical example, Baskett found through his study of neurology at Toronto General Hospital (TGH) that: “There is no mention of psychotherapy in any of the records from the Nervous Ward” [15].

In psychiatry, Ernest Jones of Freud’s inner circle was recruited to the Toronto Asylum/Hospital in 1908 as a clinician and medical school instructor —distinctly within the model of Kraepelin, with whom he had sought training for securing this appointment. Freud was regarded as a pariah by Jones’s superiors and colleagues, hence Jones was dogged by suspicion through his well-known association with Freud. Jones was directed to avoid references to psychotherapy and Freud in his medical teaching, and the archival evidence of a medical student’s detailed, 1912 class notes taken during Jones’s lectures indicate that he did indeed refrain [16]. He was, however, less discrete in addressing his peers. Brown concluded that: “Canadian physicians were to get their Freud pure and undiluted, with the ‘sexual core’ perhaps all too prominently displayed.” Hence Jones reluctantly but permanently departed Toronto in 1913. As he later wrote, “the spark died out” [17].

Hope for AN sufferers had by then emerged from a different direction. Lasègue, Gull and Charcot had piqued the imagination of another emerging medical icon who would become highly influential in his own right, Professor William Osler. Michael Bliss noted that Osler’s reading of “Charcot’s *Lectures on Localization of Regions of the Brain* had stimulated his interest in the brain’s pathology.” Then, in the summer of 1890, he “saw Charcot… at work in Paris” [18•]. With customary thoroughness, through his 1892 text, *Principles and Practice of Medicine* that affected all of Western medicine, Osler made reference to, and expanded on Gull’s AN formulation. Influenced by neurologists Charcot, Freud, and his Johns Hopkins colleague, Weir Mitchell (but contrary to Adolph Meyer, who rejected the mind-body dualism of this and other “functional disorders” such as neurasthenia [19]), Osler set down a psychogenic definition for Hysteria as: “A state in which ideas control the body and produce morbid [pathological] changes in its functions (Möbius).” Osler located AN as a crucial variant, as follows:

“The most striking and remarkable digestive disturbance in hysteria… Nothing more pitiable is to be seen in practice than an advanced case of this sort.… Although the condition looks so alarming, these cases, when removed from their home surroundings and treated by Weir Mitchell’s method [a rest and restorative regimen], sometimes recover in a remarkable way. Death, however, may follow with extreme emaciation. In a fatal case under my care the girl weighed only 49 lb. No lesions were found post mortem” [20].

A few pages along, Osler recommends —concerning hysteria generally: “To treat hysteria as a physical disorder is, after all, radically wrong. It is essentially a mental and emotional anomaly, and the important element in the treatment is moral control.” This must lead directly to inpatient status to employ “the special value” of the Weir Mitchell method (1121). In subsequent editions, drawing upon Pierre Janet’s 1905 lecture on “hysterical anorexy”, within his renowned series of fifteen psychology lectures at Harvard Medical School, Osler acknowledged that: “Hysterical patients may become insane and display persistent hallucinations and delirium, alternating perhaps with emotional outbursts of an aggravated character. For weeks or months they may be confined to bed, entirely oblivious of their surroundings… the patients must be incessantly watched, as a suicidal tendency is by no means uncommon” [21].

Osler’s widely influential text thus brought forward a potential to advance the historically desperate yet murky predicament of AN sufferers, through a number of helpful confirmations and clarifications: AN could indeed be a distinct disorder, not simply a symptom or a manipulative device; it lay squarely within medicine’s broad ambit for diagnosis and treatment; specifically, it had recently been studied and articulated by leading neurologists, noting characteristics that coalesced with their sub-discipline; the illness course was usually lengthy; and the outcome was frequently unsatisfactory and even fatal. As a category of “hysteria,” the affliction was psychic in origin and hence should be addressed through combining physical and emotional modalities – the “Weir Mitchell cure”, and talk therapies.

But what befell the promise of Osler’s elegant and widely disseminated synthesis? Let us resume examining the Toronto medical community as arguably a representative example of the front rank, documented as “excellent” by the landmark, 1910 Flexner Report [22]. It is also a centre where vital developments for AN took place at key junctures, commencing from the late 1930s.

There and elsewhere, the follow-through after the fruitful two decades from Lasègue and Gull through Charcot, Freud’s inception, and Osler’s landmark text appears to have been slight. In the 1890s, neurology and asylum psychiatry as sub-disciplines at Toronto continued to lack a vision for research. Freud’s Vienna School of psychotherapy demonstrated an interest in AN (Table 1), but they were regarded from the outset as a rival clinical modality. Professor Garfinkel observed that, in general: “At the beginning of the 20th century, anorexic girls and women faced outright hostility from the medical profession” [23••]. Neurology and psychiatry displayed only a slight interest from the 1890s in the broad delineation of “hysteria”—the nosological classification within which Osler conceptually located AN and other variants of psychical mind-body issues. In that era at Toronto, those two specialties diagnosed only a small number of patients within the category of hysteria, and none were found specifically with AN.

In the early 1890s as neuro-psychiatry emerged within medicine’s sub-discipline of neurology, Toronto Medical graduate Campbell Meyers returned after qualifying in Europe, and at Johns Hopkins, as a “friend and collaborator” of Weir Mitchell [24]. Meyers operated his own private hospital, 1893 to 1927, for the “Functional Nervous Diseases [especially] Neurasthenia or Nervous Exhaustion” [25]. From our literature search, in none of his career publications did Meyers make reference to anorexia or AN, although some sufferers from prosperous circumstances may have been admitted among his private hospital’s limit of just 20 patients at one time (with Meyers and his assistant also in residence). That facility’s records were not retained.

Meyers also held public hospital appointments, initially at St. Michael’s Hospital and then at Toronto General Hospital (TGH), 1906 – 1911, where “he operated the first public general hospital psychiatric unit in Canada” known as the “Nervous Ward” [26]. AN was by then clearly defined as “a condition marked by loss [*“an”]* of appetite [“*orexis]* with loss of weight, accompanied by delusions and marked hysterical symptoms.” “Bulimia” was then regarded as a symptom—without reference to vomiting—of “a voracious appetite” (similar to Hyperorexia, “an insatiable appetite”); and a decade later as “excessive, morbic hunger” [27].

In Meyers’s initial Report to the Superintendent on the first 16 months for the Nervous Ward, of the 69 patients who were treated and discharged, “64 suffered from Neurasthenia, 2 from Hysteria, 2 from Catalepsy [and] 1 from Epilepsy” [28]. As an example of one of this ward’s first two “Hysteria” patients, Martha G. was admitted in December, 1906. Canadian-born, age 25, a dressmaker; she complained of soreness in the back of her head and the small of her back, and headaches. Martha, whose digestion was “fair”, was discharged after 3 months. As noted, TGH records list the official diagnoses for all medical wards during the 20-year period of the Nervous Ward, to 1926. There is no diagnosis entered as anorexia or AN [29].

In corroboration, Geoffrey Reaume studied a random sample of 73 Nervous Ward patients during Campbell Meyers’s stewardship, 1906–1911 [30]. Through his detailed inspection of their charts, Reaume found 13 patients who had “declined food” for varying lengths of time, or “refused to eat”. None was assigned a diagnosis of AN. This behaviour was recorded as a regressive episode in the illness course for 9 of the 13 who had *not* refused to eat prior to admission, and who returned to eating normally before discharge. For the other four patients, however—single women between ages 17 to 41—“the refusal to eat food was noted as a sign of potentially destructive pre-admission behaviour”. Reaume felt that some of this and other uncooperative behaviour on the ward could be discerned as manipulative, while at other times, “patients were not devising a strategy but reacting to their immediate situation. Thus patient resistance could be sporadic and spontaneous or more calculating.” When genuine (not feigned), it may have been symptomatic of their illness or symbolic of real fear or anger, perhaps relating “to traumatic episodes from the past, such as physical abuse and personal rejection” [31].

Turning to Toronto’s institutional sector of asylum-based “alienism” or “mental diseases” [32], we find a pattern consistent with patients’ diagnoses recorded for that era’s general medical wards and neurological ward at TGH. Toronto’s “Hospital (formerly Asylum) for the Insane” also reported no admissions explicitly for AN [33].

Notwithstanding, Dr. Daniel Clark, Superintendent (1875–1905) of the Toronto Asylum, is remembered as an advocate for efficacious clinical treatment for women. Clark opposed the gynaecological surgery that was prescribed and carried out to treat women’s mental disorders, as directed by his counterpart at the London (ON) Asylum, R.M. Bucke (celebrated for his links to the poet, Walt Whitman). Ultimately, their Provincial overseers ordered Bucke to desist. Clark had earlier registered his objections in publishing the first Canadian psychiatry text for medical students, declaring that:

“Uterine and ovarian disturbances and diseases do not produce insanity to the extent supposed [citing eminent gynaecological support]. It is a matter of regret that modern surgery, which has made such advances during the past decade, has unsexed so many women because of slight troubles in one or both ovaries, under the impression that they are the cause of many nervous diseases and mental troubles” [34].

This 1895 textbook, however, now seems inconsistent in that Clark omitted any reference to anorexia and AN. Moreover he offered just two passing and relatively dismissive references to hysteria, noting in the first that it and two other conditions were “too temporary, evanescent or intermittent to constitute psychic disease” [35].

Soon after Clark’s era, from 1913, the distinguished American psychiatrist-psychotherapist, William A. White’s *Outlines of Psychiatry* [36] dominated as a North American-wide text, through nine editions. It was one of two psychiatry texts at the U. of Toronto Medical School, along with Ross Diefendorf’s *Clinical Psychiatry*, that were based on Emil Kraepelin’s classifications [37].

Our inspection of White’s text, based on his 4th and 9th editions, indicates that, as with Clark’s text, no explicit reference was made to anorexia nor AN. White included less than five pages on hysteria, as part of his “Borderland and Episodic States” chapter, while opining that: “The most important theory of hysteria today is that of Freud” [38]. In his inconclusive discussion of “Hysterical Insanity/ Psychoses”, White may well have had AN in mind while grappling for “an explanation for the association of physiological disturbances with hysteria, such as *the false* gastropathies [diseases of the stomach]” [39]. Ironically, however—for a chapter on “Treatment”—White includes this distinctly non-empathic and rather testy guidance concerning: “**Refusal of Food**.– This is one of the most *annoying symptoms* met with[,] and yet is quite common among the depressed cases.” Moreover: “There are many methods of artificial feeding, but the method of tube-feeding is the only one that merits much attention.” White continues by providing detailed clinical steps for administering restraint and tube-feeding [40].

## Simmond’s Disease Versus Psychiatric Alternatives—1914 to 1939

We have observed that, at Toronto as representative of major centres, Osler’s classification of AN as a category of Hysteria initially found little traction within biological medicine (neurology and psychiatry excluding Freud and Janet)—evidently failing to overcome a stigmatic antipathy toward addressing it. Then, as Garfinkel relates: “It was in this setting of hostility and mistrust to starving young women that a twist was added” [41]. Stigma was likely reduced and clinical interest was certainly piqued in 1914 by an alternative—and distinctly biological—explanation for AN, as a disorder of the pituitary gland. As Bell summarized, for the next quarter-century “medical opinion on the causes of self-induced starvation changed dramatically”. Autopsy findings by Morris Simmonds of Hamburg showed “destructive lesions of the pituitary gland in a severely-emaciated pregnant woman”. From there, “most physicians treated virtually all presentiments of extreme malnutrition, even if apparently self-induced, as primary endocrine disturbances” [42].

Although the Simmonds disease narrative was widespread, encompassing influential clinician-researchers such as at the Mayo Clinic (where “physicians defined and treated anorexia nervosa as if it were a general metabolic disorder” [43]), that view had been disputed over many years by biological psychiatry. The Henderson and Gillespie text, which had begun to succeed William White’s from 1927, included in its 1933 edition no mention of Simmonds, pituitary, thyroid or other glandular connections to AN. They identified AN in the Oslerian tradition as a “visceral symptom” [i.e., appearing in body organs] but without a structural lesion, hence grouped among psychoneuroses – hysteria in particular – and “one of the commonest and most interesting” [44].

In 1930, as further evidence that biological-institutional psychiatry at Toronto had not adopted the Simmonds disease premise for AN, a course of instruction for psychiatrists, psychologists and social workers who would operate Mental Health Clinics as an adjunct to the Provincial Psychiatric Hospitals received a lecture on “Pituitary Disturbances”; no reference was made to Simmonds or AN [45]. Subsequently, in 1936, Prof. C.B. Farrar, Head of Psychiatry at Toronto, emphasized in his synopsis for the undergraduate medical program in psychiatry, “that the so-called ‘functional’ or ‘nervous’ conditions, as reflected in the patient’s thinking and feeling states, are strictly psychiatric problems, whether the question be one of kind or degree” [46].

Then in 1938, two U. of Toronto internists, Ray Farquharson and Herbert Hyland, comparatively studied Simmonds disease and AN. Their publications described the latter “as a mental disorder in its own right and not a disorder of the endocrine gland” [47•]. Joan Blumberg’s detailed analysis of this and other somatic experimentation concludes: “By 1940 explanations that ‘blamed’ the thyroid, ovaries, pituitary or pancreas were less than satisfactory because anorexia nervosa was being reconstructed as a ‘psychologic disorder’ by mid-twentieth-century psychiatry” [48].

## The Early DSM Era

When the DSM-1 appeared in 1952, Simmonds disease (by then recognized as *not* being psychiatric, among the other considerations noted) was not included. AN was listed as: [49]

“006-580 Psychophysiologic gastrointestinal reaction”

This broad category included such specified types of gastro-intestinal disorders as peptic-ulccr-like reaction, chronic gastritis, ulcerative or mucous colitis, constipation, hyperacidity, pylorospasm, heartburn, irritable colon, together with “anorexia nervosa” and others in which emotional factors play a causative role.

The DSM-2 (Second Edition), appeared in 1968 with this *remainder* or *all-other* style of listing: [50]

**“VH. SPECIAL SYMPTOMS (306)**

**306 Special symptoms not elsewhere classified**

This category is for the occasional patient whose psychopathology is

**48 MENTAL DISORDERS**

manifested by a single specific symptom. An example might be anorexia nervosa under *Feeding disturbance* as listed below. It does not apply, however, if the symptom is the result of an organic illness or defect or other mental disorder. For example, anorexia nervosa due to schizophrenia would not be included here.”

The Henderson and Gillespie text’s 1969 edition was thus on firm ground in retaining AN within Hysteria, although that group’s larger classification, “essentially a clinical one”, was now designated as anxiety neuroses. Significantly, the locus of clinical treatment was now clarified: “Cases of anorexia nervosa should be admitted to the psychiatric unit of a general hospital”. Also significantly, in conjunction with certain physical treatments that subsequently were replaced: “*Psychotherapy* is the main form of treatment of neurotic reactions…. When the patient’s physical state no longer causes anxiety, psychotherapy should commence” [51].

## Conclusion: Anticipating the Impacts of Dr. Hilde Bruch

The late nineteenth to twentieth centuries’ “silo” approach to medical specialization often resulted in a rival compartmentalizing of illnesses – sometimes leading to ambiguity or worse for sufferers afflicted by inexplicable symptoms, as with AN. As medicine gradually adapted routines for resolving responsibilities, psychiatry’s understanding of, and armamentarium for AN began to evolve by mid-century toward an interdisciplinary, multidetermined patient-centred focus.

Professor (Psychiatry and Psychotherapy) Hilde Bruch taught charismatically at Baylor College of Medicine and wrote two important monographs launching hope for AN sufferers [52]. She also inspired the imaginations of others who, like herself, dedicated their careers to combating what became a pandemic. Along with inexplicable increases in the morbidity and mortality of AN, the intractability and severity of individual suffering persisted.

Doing justice to Dr. Bruch’s contributions, and to the scientific advancements that have been made and continue to be pursued, will require more scope and space than is available here. Hence, even a summation of her career and its era must await future scholarship by historians well versed in the sophisticated science that has evolved, and continues to do so.

Vital to those next chapters will be the contributions of legendary healer-scientists who have absorbed and carried forward Hilde Bruch’s elegant teaching, notably: Professor Gerald F.M. Russell (London, UK) with Drs. George Szmukler and Janet Treasure [53••]; and (full disclosure—one of our favourite mentors) Professor Paul E. Garfinkel (Toronto) [54••]. Thankfully, these and other outstanding leaders from the first generation who were mentored and inspired by Dr. Bruch continue to guide this field.

## Footnotes

1 Historically, our understanding of EDs evolved from symptoms, e.g. anorexia, bulimia, obesity, to today’s DSM/ ICD cluster of discreet disorders, notably anorexia nervosa (AN), bulimia nervosa (BN) and binge eating disorder
